# Gastric xanthelasma associated with hyperplastic polyp and mucosal erosions: report of an unusual case and literature review

**DOI:** 10.1093/omcr/omy051

**Published:** 2018-08-11

**Authors:** Malak Alzahrani, Areej Alqunaitir, Mohammed Alsohaibani, Ammar C Al-Rikabi

**Affiliations:** Department of Pathology, Histopathology Unit, King Saud University, King Khalid University Hospital, Riyadh, Saudi Arabia

## Abstract

Gastric xanthelasma is a rare benign tumor-like lesion which is usually observed as an incidental finding due to its asymptomatic presentation. Grossly, it is a well-demarcated yellow-white plaque which is microscopically formed by clusters of foamy macrophages in the lamina propria. The pathogenesis and clinical significance are not clear. Gastric hyperplastic polyps are rarely associated with xanthelasma. Mucosal erosions also appear to have an association with the combined lesions of hyperplastic polyp and xanthelasma. Here, we report a rare case of simultaneous occurrence of gastric xanthoma with hyperplastic polyp and mucosal erosions. The lesions are observed in a 78 years old male who presented with a history of chronic anemia. The histological features together with a literature review of other similar reported cases are described and compared.

## INTRODUCTION

Xanthelasma/Xanthoma also known as lipid islands are uncommon gastrointestinal tract (GIT) features and the stomach is the most common site of reported upper GIT lesions [1]. Gastric xanthomas (GX) are rare, non-neoplastic incidental findings with an incidence ranging from 0.2 to 0.8% [1]. The pathogenesis and clinical significance of GX are not clear [2]. Endoscopically, these lesions usually present as well-demarcated round, yellow-white plaques. Microscopically, GX is characterized by collections of foamy macrophages in the lamina propria [2]. Histological confirmation of GX is mandatory because its appearance may mimic malignancy and once the histological diagnosis of GX is confirmed, it is necessary to look for accompanying premalignant conditions such as chronic gastritis, *Helicobacter pylori* associated gastritis, intestinal metaplasia, atrophic gastritis and gastric dysplasia [2].

Hyperplastic polyps represent the most frequent type of gastric polyps with a reported incidence of 46% [3]. Only few cases of coexistence of gastric hyperplastic polyp and xanthelasma have been reported to date [4]. The extremely rare coexistence of these two lesions in a patient with erosive gastritis has also been reported in 1989 for the first time by Lin *et al*. [5]. In this report, we describe the case of a 78-year-old male who presented with a combination of GX, mucosal ulceration and hyperplastic polyp.

## CASE REPORT

**Figure 1:**
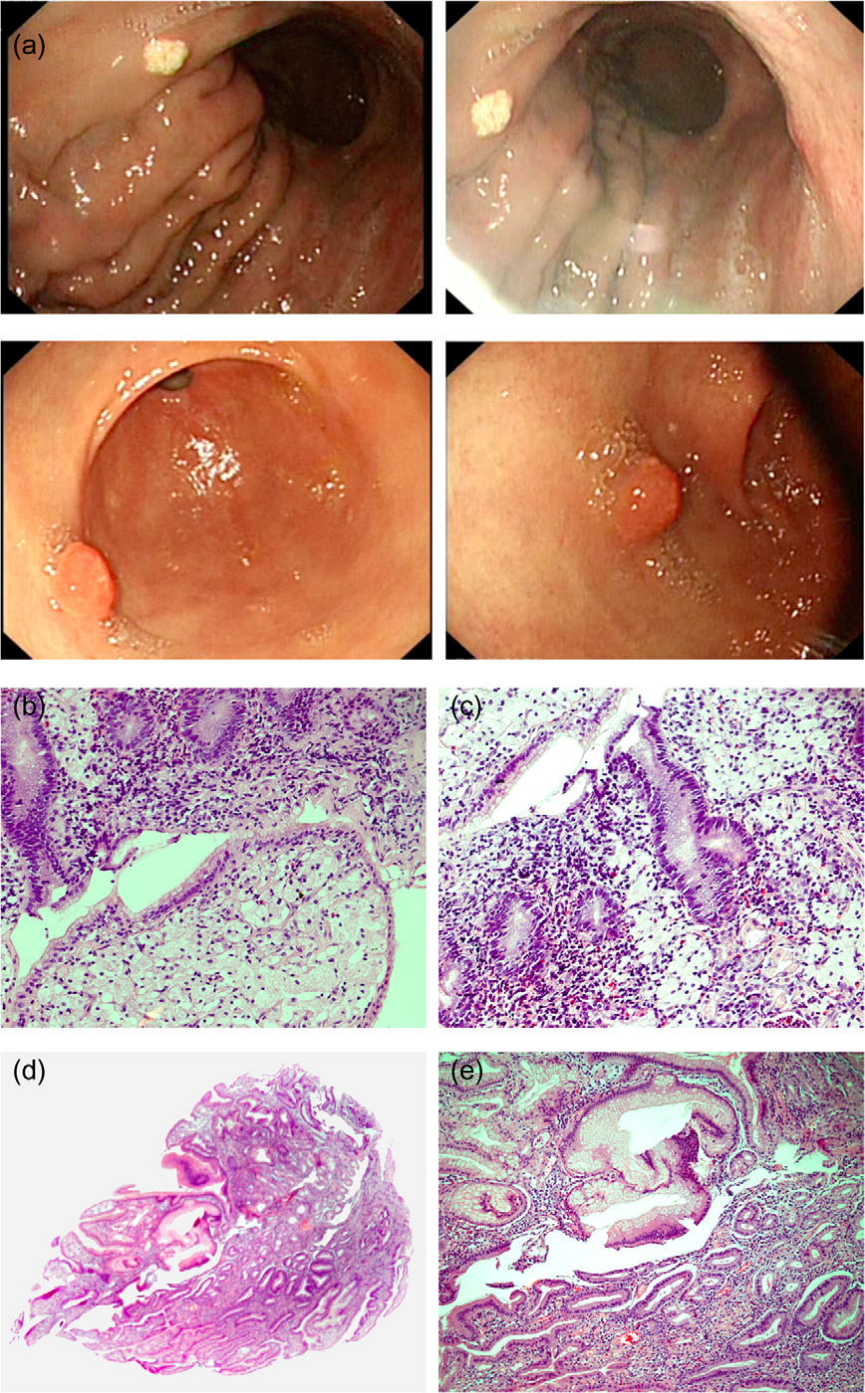
(**a**) Endoscopic images of gastric lesions. The upper two endoscopic images showing the same yellow-white gastric lesion at the gastric fundus measuring 0.7 cm. The lower two endoscopic images showing a single pedunculated gastric polyp at the antrum measuring 1.0 cm. (**b**) Photograph of the gastric biopsy showing a large aggregate of foamy macrophages with adjacent inflamed mucosa (H&E stain ×200). (**c**) Inflamed gastric mucosa showing numerous foamy macrophages consistent with xanthoma in the gastric lamina propria. Note the presence of active acute on chronic gastritis in the adjacent gastric glands (H&E stain ×200). (**d**) Low power microscopic view of gastric polyp (H&E stain ×40). (**e**) Inflamed gastric hyperplastic polyp containing cystic glands lined by clear Parietal cells. Note the presence of goblet cells in the upper left side of the picture which is indicative of intestinal metaplasia (H&E stain ×100).

A 78-year-old male presented with symptoms of chronic anemia. His physical examination showed bleeding per rectum. Blood investigations revealed iron deficiency anemia. The cause of anemia was fully investigated including CT abdomen/pelvis, upper and lower GI endoscopy. Gastrointestinal malignancy was excluded. Colonoscopy showed anal hemorrhoids. His anemia was secondary to chronic blood loss from anal hemorrhoids. Interestingly, esophagogastroduodenoscopy showed an incidental findings including yellow-white gastric lesion at the gastric fundus measuring 0.7 cm and a pedunculated gastric polyp at the antrum measuring 1.0 cm (Fig. 1a). Histological examination of the gastric biopsy specimens revealed an inflamed and hyperplastic polyp at the antrum (Fig. 1d and e). The fundus showed that the mucosal lamina propria contained chronic inflammatory infiltrate and clusters of oval shaped cells with abundant, foamy cytoplasm consistent with the diagnosis of GX (Fig. 1b and c). Biopsy from the surrounding area of the GX lesion showed a significant amount of acute on chronic inflammatory cells infiltration suggestive of severe gastritis with mucosal erosions and intestinal metaplasia (Fig. 1e) associated with a small number of *H. pylori*-like organisms. There was no evidence of glandular dysplasia or malignancy in the sections examined. The gastric lesions did not seem to be contributing to his anemia and therefore was not resected. The anemia was resolved after ligation of anal hemorrhoids and 3 months course of oral iron supplement.

## DISCUSSION

Gastric xanthomas are rare and non-neoplastic incidental findings. The majority of case reports agreed that GX mostly occur in the antral region [1], however, the case that we have encountered was located in the fundus of the stomach. Although GX can be seen in all age groups, it is considered to be more prevalent in older patients. In our case, the lesion was diagnosed in a 78-year-old male which is in keeping with age-range mentioned in the literature [6].

The aetiology, pathogenesis and clinical significance of GX in relation to other gastric diseases remain unclear [2]. It is believed that the gastric mucosa affected by certain pathological processes is more prone to develop GX and for this reason, it is mandatory to look for other accompanying premalignant conditions [2]. The close relationship between *H. pylori* infection and GX has been described in various studies. Isomoto *et al.* reported a close relationship between GX, *H. pylori* infection and atrophic gastritis and showed that the prevalence of *H. pylori* infection was significantly higher in patients with GX compared to patients without GX [7]. *Helicobacter pylori* infection has been found to present on the surface of epithelial cells in 48% of biopsy specimens of GX [8]. GX appears to be related to healing processes in response to tissue damage and is provoked by inflammation induced by *H. pylori* infection and foamy transformation of macrophages is secondary to phagocytosis of the bacteria penetrated into the lamina propria [7]; therefore, *H. pylori* infection is believed to be an aetiological factor for GX formation. In our case, *H. pylori*-like organisms were observed in small amount by histopathological examination.

Koksal *et al*. [9] conducted an observational case–control study and they reported an association between multifocal atrophic gastritis and/or intestinal metaplasia with the presence of gastric xanthelasma. In our case, intestinal metaplasia was detected by histopathological examination. It has been also showed that GX may serve as a warning sign for the presence of gastric malignancy [10]. However, there was no evidence of dysplasia or malignancy in our case.

Coexistence of GX and hyperplastic polyp is rarely mentioned in the English literature, with only eight cases reported to date [5, 11–13]. Furthermore, our case has another extremely rare coexistence of GX, hyperplastic polyp and mucosal erosions. Our literature search showed that only one reported case of similar association was recorded and published by Lin *et al*. [5].

In this context, GX formation is thought to be secondary to an inflammatory reaction to a focal mucosal damage. The histological confirmation of GX diagnosis is advisable in order to rule out the possibility of gastric malignancy or any precancerous lesions of gastric mucosa which is frequently associated with this lesion.

## Conflict of Interest

No conflict of interest.

## Guarantor

Malak Alzahrani.
